# GraphoGame – a catalyst for multi-level promotion of literacy in diverse contexts

**DOI:** 10.3389/fpsyg.2015.00671

**Published:** 2015-06-10

**Authors:** Emma Ojanen, Miia Ronimus, Timo Ahonen, Tamara Chansa-Kabali, Pamela February, Jacqueline Jere-Folotiya, Karri-Pekka Kauppinen, Ritva Ketonen, Damaris Ngorosho, Mikko Pitkänen, Suzanne Puhakka, Francis Sampa, Gabriel Walubita, Christopher Yalukanda, Ken Pugh, Ulla Richardson, Robert Serpell, Heikki Lyytinen

**Affiliations:** ^1^Agora Center, University of Jyväskylä, JyväskyläFinland; ^2^Department of Psychology, University of Jyväskylä, JyväskyläFinland; ^3^Niilo Mäki Institute, JyväskyläFinland; ^4^Department of Psychology, University of Zambia, LusakaZambia; ^5^Department of Educational Psychology and Inclusive Education, University of Namibia, WindhoekNamibia; ^6^Department of Teacher Education, University of Helsinki, HelsinkiFinland; ^7^Department of Education, Sebastian Kolowa Memorial University, LushotoTanzania; ^8^Read to Succeed, LusakaZambia; ^9^Department of Educational Psychology, Sociology and Special Education, University of Zambia, LusakaZambia; ^10^Zambia National Union of Teachers, LusakaZambia; ^11^Haskins Laboratories, Yale University, New Haven, CTUSA

**Keywords:** GraphoGame, reading intervention, game-based learning, mobile technology, literacy, Africa

## Abstract

GraphoGame (GG) is originally a technology-based intervention method for supporting children with reading difficulties. It is now known that children who face problems in reading acquisition have difficulties in learning to differentiate and manipulate speech sounds and consequently, in connecting these sounds to corresponding letters. GG was developed to provide intensive training in matching speech sounds and larger units of speech to their written counterparts. GG has been shown to benefit children with reading difficulties and the game is now available for all Finnish school children for literacy support. Presently millions of children in Africa fail to learn to read despite years of primary school education. As many African languages have transparent writing systems similar in structure to Finnish, it was hypothesized that GG-based training of letter-sound correspondences could also be effective in supporting children’s learning in African countries. In this article we will describe how GG has been developed from a Finnish dyslexia prevention game to an intervention method that can be used not only to improve children’s reading performance but also to raise teachers’ and parents’ awareness of the development of reading skill and effective reading instruction methods. We will also provide an overview of the GG activities in Zambia, Kenya, Tanzania, and Namibia, and the potential to promote education for all with a combination of scientific research and mobile learning.

## Introduction

The widespread endurance of illiteracy across the world deprives millions of citizens of economic and political opportunities to secure their basic human rights. It is estimated that almost one in six people over the age of 15 cannot read and write, and most of them live in low-income countries where only a minority of children have access to quality basic schooling (Campaign for Education, 2015). In order to tackle this challenge, it is useful to see what has been done in countries with successful education systems, and how the experience from these countries could be used for the benefit of others. In this article we show one example of such a process.

The national education system in Finland has received international recognition. Literacy has always been highly valued in Finnish culture. Anecdotally, almost from the time of establishment in the 16th century of the Finnish orthography, a good incentive for learning to read was the fact that people were not permitted to marry without first passing a public literacy examination administered by the local priest. In present day Finland, the majority of school children learn the basic reading skill within the first months of school entry. Failure to learn to read at the same time as classmates can be a personal tragedy that affects self-esteem and leads to long-term negative consequences, such as depression, behavior problems, and other mental health issues (Mugnaini et al., 2009; Undheim et al., 2011). Since identification of the children at risk for learning difficulties was of high importance to Finnish society, the Academy of Finland provided funding for the Jyväskylä Longitudinal study of Dyslexia project (JLD), starting in 1992, which has followed children with familial risk for dyslexia from birth to early adulthood. JLD project increased our knowledge of the developmental patterns that predict dyslexia and thus helped us to develop methods to identify children who are likely to face difficulties in their reading acquisition. However, identifying a problem has little value without the means to support children to overcome the problem, or better still, to prevent the problems from occurring by preventive training offered at an early stage. For this purpose, the JLD team was able to develop (with the support of, e.g., the Ministry of Education and Culture of Finland) ways to support at-risk children’s reading acquisition. From the outset, the goal was to develop an individually adapted training regimen which would motivate children to train until they reached the skill level of their typically developing peers and which would be accessible, without charge, to all children in need of training.

Jyväskylä Longitudinal study of Dyslexia project taught us that the key to achieving basic literacy skill in the alphabetic script of Finnish language is to learn how letters (graphemes) and sounds correspond to each other in the writing system (e.g., Lyytinen et al., 2005, 2009). Children with problems in reading acquisition appear to have problems in differentiating and manipulating speech sounds and consequently, in connecting these sounds to matching letters (Lyytinen et al., 2009). There was a need to find a motivating, engaging way of training letter-sound connections and reading skills. At the beginning of the 21st century, there was something that all the children were motivated to spend their time with: computers. A computerized learning game for Finnish learners was developed, and controlled intervention studies showed from the beginning that playing the game improved performance in literacy-related tasks (e.g., Hintikka et al., 2005; Lyytinen et al., 2007; Saine et al., 2010, 2011; Lovio et al., 2012; Heikkilä et al., 2013). For instance, the intervention by Heikkilä et al. (2013) was acknowledged for its high quality in a meta-analysis of intervention research of reading difficulties (Galuschka et al., 2014). Shortly afterwards, an international study with EU’s Marie Curie Excellence funding of four European countries was started. International adaptations of the game have also proved to be effective, for example, the impact of German GraphoGame (GG) on decoding performance and associated brain activity was demonstrated with brain event-related potential (ERP) and functional magnetic resonance imaging (fMRI) data (Brem et al., 2010). Long-term effects of GG training have been observed by Saine et al. (2010, 2011), who found that poor readers who received remedial education including regular GG sessions in the first grade, still outperformed the children who had received remedial education without GG in the beginning of third grade. Follow-up effects were also seen 4 months after the intervention period in the English game version experiment (Kyle et al., 2013).

Earlier research has shown that technology has the potential to prevent and remediate reading difficulties. A research synthesis by Blok et al. (2002) suggests that computers have a positive but small effect (*d* = 0.19) in supporting the instruction of beginning reading skills. A more recent review (Cheung and Slavin, 2013) with more rigorous standards for included studies also found educational technology to have a small but significant effect (ES = 0.14) in the support of struggling readers. In this review, educational technology was defined to include not only computer-based approaches, but a variety of electronic tools, such as the use of video or multimedia as components of reading instruction. Still today, the use of digital game -based approaches in the remedial reading instruction is still rare. Results of a recent meta-analysis (Wouters et al., 2013) suggest that generally, digital games are more effective than conventional instruction methods in terms of learning (*d* = 0.29, *p* < 0.05), but not necessarily in terms of motivation (*d* = 0.26, *p* > 0.05). Because of lack of systematic research concerning the use of serious games in beginning/remedial reading instruction, conclusions as to the overall effectiveness of games in this area of learning can be drawn.

**FIGURE 1:**
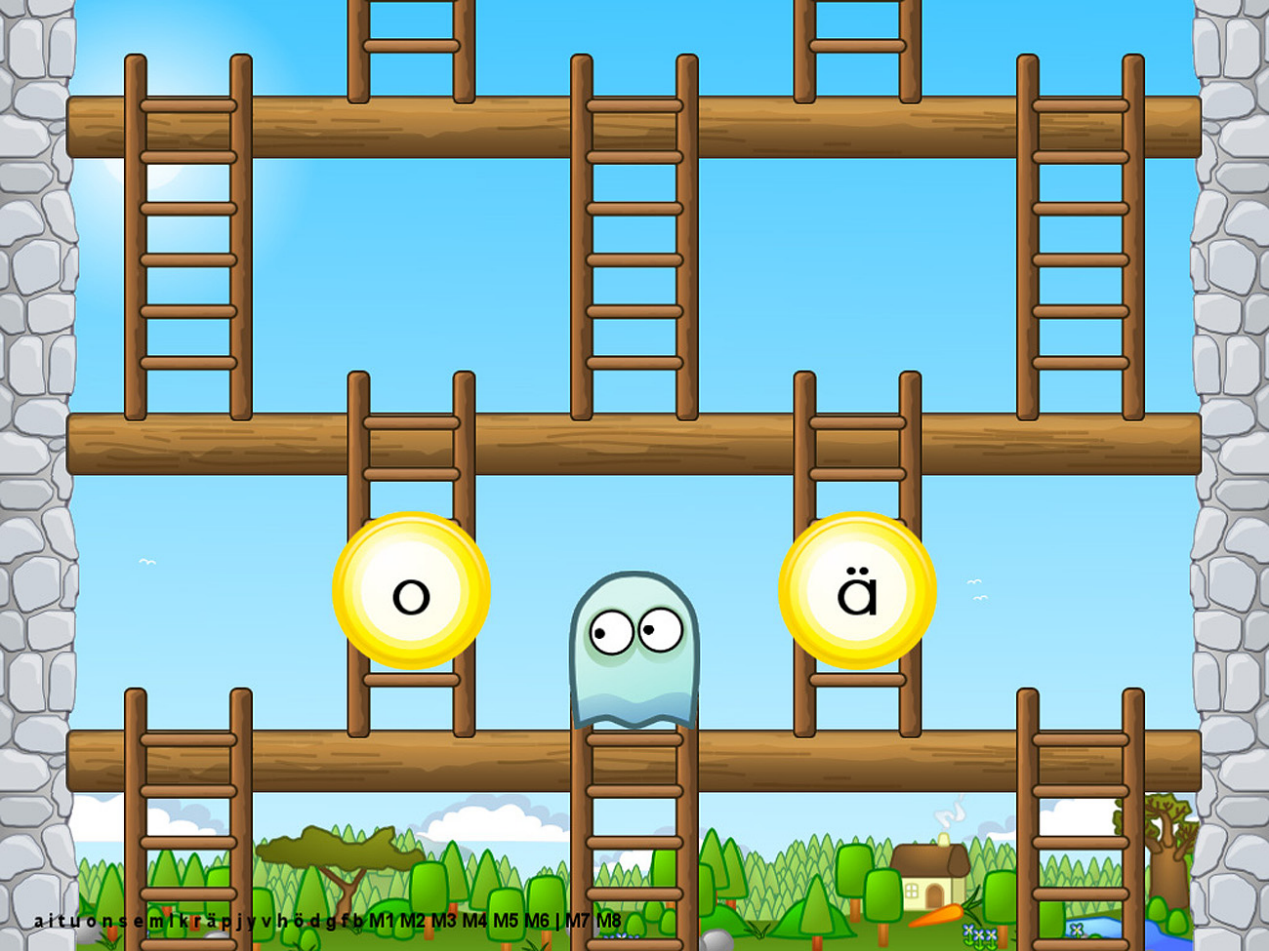
**A screenshot of a GraphoGame (GG) task in which the child is expected to choose the letter matching the letter sound**. In case of a correct response, the game character climbs the ladder and falls down the ladder when the response is incorrect.

Today, GG^1^ is a web-based scientifically validated, non-profit internet service for reading acquisition that is already used widely in Finland on multiple platforms. GG teaches the learner basic literacy skills and at the same time, collects data on the learning process for research and education purposes (Richardson and Lyytinen, 2014). Pedagogically, GG teaches children grapheme-phoneme connections based on synthetic and analytic phonics instruction by constantly adapting to the player’s skill level. The adaptation technique aims to keep the training optimally challenging for the child, so that the child receives mostly positive feedback (around 80% of trials). Optimal level of challenge is one of the crucial features of engaging learning environments (Ryan et al., 2006), and the high amount of positive feedback is expected to maintain the child’s positive self-concept as a learner. Children’s self-reports suggest that they enjoy playing GG, and also parents have observed that their children are highly motivated and concentrate well while using GG (Ronimus et al., 2014). A screenshot of a GG task can be seen in **Figure 1**.

The Finnish versions of GG (Ekapeli) are provided via the LukiMat service^2^ which is maintained by the Niilo Mäki Institute and sponsored by the Ministry of Education and Culture. The game is targeted at children who evidence early signs of reading difficulties (such as difficulty in learning letter names) at the end of kindergarten (6 years of age) and at children who are in Grades 1 or 2 at school and require support with basic decoding skills or reading fluency. Since the launch of the LukiMat service in 2008, GG has acquired approximately 270,000 registered players in Finland, and every day, around 6000 children log on to play. As each age cohort has about 60,000 children in Finland, it seems that the game is in great demand, even in the country with one of the top literacy scores in the OECD countries (OECD, 2010).

### Biological and Environmental Factors behind Reading Problems

A major obstacle to increasing our understanding of learning difficulties is that the vast majority of information on the topic comes from Western societies and describes learning as it happens within high-income societies. What is currently known is that from the cognitive and neurobiological perspectives, the most important predictive factors emanate from the development of spoken language skills from very early on. The JLD results show that such individual factors based on genetic starting points reveal the first observable indications during the first days of life (Guttorm et al., 2005, 2010; Leppänen et al., 2012). Such innate factors affect the whole development before the time that children start learning to read. Environmental factors also play a role in the manner in which the genetic predisposition manifests itself in children’s development. The models of reading presented by the parent who has severe difficulties create an environment that may, for example, affect the child’s motivation to read. Our genetic variation, which makes us better in some abilities and and less so in other domains, tends to impact in such a way that we tend to avoid exposing ourselves to activities or hobbies where our mastery is compromised. Thus, it is very likely that a child who finds it difficult to manipulate spoken language in language play and struggles with learning to read, will start to avoid such activities (Onatsu-Arvilommi and Nurmi, 2000; Poskiparta et al., 2003). In fact, such a tendency toward avoidance was observed as the most important factor that needs to be prevented before children can overcome their problems in reading acquisition (Eklund et al., 2013).

Nonetheless, on a global level, the role of biological factors is insufficiently studied. Many environmental factors environmental factors, such as malnutrition and early childhood illness may have an effect on learning abilities. The poor quality of education itself is a major risk factor. Inadequate teacher training and negative attitudes in the community play a role, and most of all, the language of instruction may be an underestimated cause of poor learning outcomes. While the use of indigenous languages as media of instruction has been defended on the basis of human rights and preservation of local cultures, the impact of language goes much deeper. The relationship between the spoken language and the writing system has a large effect on the literacy learning rate. The amount of written symbols in the writing system and the rules of connecting them to the speech sounds vary across languages. The easiness of learning to read is related to the amount of letter-sound pairs learner needs to memorize. One cannot learn to connect spoken to written language without that the learner having at least implicit awareness of the speech sounds of the language. In certain languages, such as English, sounding out some items (e.g., exception words such as YACHT) is difficult since these words violate spelling to sound rules and thus pronouncing them correctly requires access to the meaning and/or derivation of the word. In contrast languages such as Finnish can be fully transparent: letter to sound associations always produce the correct pronunciation. These differences between languages have profound effects on learning (e.g., Aro and Wimmer, 2003; Seymour et al., 2003).

Understanding how different languages affect the rate of reading acquisition has only recently come to the attention of researchers. A cross-linguistic comparison study by 14 European countries showed that it takes significantly longer to learn the foundations of reading in English in comparison to languages with more transparent orthographies (e.g., Seymour et al., 2003). This study was the first to document that the writing system (orthography) plays a major role in learning to read within the alphabetic systems. Alphabetic orthographies can be placed along a continuum with transparent and opaque being the opposites. In a completely transparent orthography such as Finnish or Italian, there is only one speech sound (phoneme) for each written symbol (grapheme) and there are no exceptions in spelling or pronunciation. At the other extreme, the English orthography is devoid of consistency in letter-sound connections: spelling and pronunciation for each word needs to be learned more or less separately. In the study by Seymour et al. (2003), Finnish children were the fastest learners due to the transparency of the orthography. The Finnish child needs to learn only 23 connections between letters and sounds and how to combine them into syllables and words. After learning the grapheme–phoneme connections, inventing the assembly and automatizing the retrieval of the connections from memory, Finnish children are able to read without problems, any pronounceable sequence of letters. Almost all Finnish learners acquire full accuracy during the first school semester. In contrast, the reader of English must master 100s of connections between spoken and written items. A substantial number of English words cannot be pronounced without seeing the whole word. Instead, the child needs to memorize certain letter sequences by heart. Thus, using English language as a universal model for reading instruction is problematic. Scientific research in literacy cannot be anglocentric or eurocentric, or founded on a single orthography (Share, 2008). Instead, the process of literacy acquisition needs to be studied in all languages and writing systems, and the instruction methods need to be developed for optimal approach for each orthography.

International organizations, such as USAID, have already taken into account the information learned about the impact of specific orthographies on reading acquisition and emphasized in the guidelines of Early Grade Reading Assessment Toolkit (2009: RTI International) that it is important to acknowledge the differences in orthographies in the development of literacy assessment tools. Consequently, the use of literacy materials that are based on the English language might not be suitable in other languages. However, most existing literature about learning to read and teaching literacy is based on research conducted in English speaking countries and may be misleading for literacy instruction in other languages. The vast majority of the world’s alphabetic languages are spelled consistently (Abadzi, 2013). In such transparent reading environments, decoding skills can be achieved in 3–4 months, in contrast to 2–3 years needed for mastery of the non-transparent English writing system (Seymour et al., 2003; Abadzi, 2013). After achieving basic decoding skills, children are able to use their reading skill for learning and must be further motivated to read in order to develop reading fluency. Reading fluency in the mother tongue lays a cognitive and linguistic foundation, not only for full literacy, but also for learning additional languages and other scholastic subjects (Ball, 2011).

In addition to biological risk factors and compromised learning environment, a third factor that affects reading acquisition, social support, needs to be considered. One of the most important ways in which parents and teachers can support their children’s literacy acquisition, is to find ways to motivate the child to become interested in reading-related activities and to start using reading skills early on. Maintaining the learner’s interest in reading is the most efficient protective factor among children at risk for reading difficulties (Eklund et al., 2013). Children must be motivated to learn to read and to continue to improve their reading skills by reading. In Western societies, an abundance of reading materials in the ordinary living environment allows for independent reading practice outside school. However, in more disadvantaged societies, there may be little else to read except school books and complex religious texts, and even those may not be plentiful.

### Literacy Crisis in Africa

In Sub-Saharan Africa, one in five children were out of school in 2011 (Education for all Global Monitoring Report [EFA GMR], 2013/2014) and across Africa, less than half of children reach the end of primary school (Heugh, 2011). School access and high drop-out rates have been major concerns in international development for a long time. It is only recently that the quality of education and the actual learning outcomes of children have received attention. From 650 million primary school age children, at least 250 million were not learning the basics in reading and mathematics (Education for all Global Monitoring Report [EFA GMR], 2013/2014). The remaining 130 million children are the true tragedy: as many as one third of children who complete primary school are unable to read a sentence (Education for all Global Monitoring Report [EFA GMR], 2013/2014). The latest statistics show that, in 17 Sub-Saharan countries, less than half of children are learning the basics and are poorly prepared for transition to secondary education (Education for all Global Monitoring Report [EFA GMR], 2013/2014). Such outcomes are the rule in Africa, not the exception (Africa Progress Panel Report, 2012).

These dramatic numbers tell us that the full scale and gravity of the global illiteracy crisis has only recently gained public attention. It is not just about how many children cannot go to school in Africa, it is the fact that millions of children are failing to learn despite years of school attendance (Africa Progress Panel Report, 2012). To prevent the growth of new generations of illiterate adults, primary classroom instruction must focus on methods to teach fluent reading in early grades, especially in countries where the dropout rate is high (Abadzi, 2004). Fortunately, improving the quality of learning is likely to be central to the post-2015 global development framework (Education for all Global Monitoring Report [EFA GMR], 2013/2014).

It is worth considering whether the poor literacy results in Africa are related to the fact that it is the only continent where the majority of children still start school using a foreign language (Ouane and Glanz, 2010). In sub-Saharan African countries, it is common that the language of instruction (English, French, Portuguese) is a second or third language to the teachers. In addition, only one third of teachers have teaching qualifications (Griffin, 2012). In most African countries, teachers are expected to teach children to read and write in a language which is both unfamiliar to the learners and in which teachers themselves may have little competence (Ouane and Glanz, 2010). The most common languages used in African education systems are English and French, amongst those alphabetic orthographies that have been shown to be the most difficult to learn to read and write – even for native speakers (Seymour et al., 2003). It would seem obvious that, whenever possible, early literacy instruction should be delivered within a transparent, consistent orthography with a small number of letter-sound pairs to be learned. The quicker children learn decoding, the earlier they can proceed toward practicing fluency and using literacy as a medium for learning.

In Africa, a quick transition from decoding to fluency is necessary since it is common that the language of instruction is English or French after Grade 4, even if the early literacy has been taught in an African local language. This is a cause for concern because it has been recommended that children should not be required to move on to second language instruction before they are fully literate and academically fluent readers in their first language, but typically, the latter does not happen at all, or may be reached by Grade 6 (Ball, 2011**).** Therefore, it is not an exaggeration to say that poor learning outcomes in Africa are intimately linked to the language factor, which seems relatively neglected in the discourse of international development (Wolff, 2011). Research into literacy acquisition in local African languages can provide significant new insights to better education. There is a serious shortage of literacy specialists in Africa (Alidou, 2011) and UNESCO has pleaded with the research community to fill the gap in knowledge related to mother tongue-based bi/multilingual early education practices (Ball, 2011). Multilingualism is and will be an integral feature of African reality, and therefore it should be taken account in educational planning. However, the interconnectedness between language, communication and effective teaching and learning is currently misunderstood outside expert circles (Wolff, 2011).

It is reasonable to expect that societies that have successfully achieved full literacy in their own education systems provide assistance to those in desperate need for evidence-based solutions. In present day Africa, mobile phones offer an affordable and easy-to-use gateway to reading material and access to the Internet gives a person more reading material than in any physical library ever built (West and Chew, 2014). The best thing about mobile phones is that most people have them: from the 7 billion people on earth, only 4.5 billion have a toilet but over 6 billion have a mobile phone (United Nations, 2013). A key strategy for the promotion of mobile reading is simply to provide people with the opportunity to try it (West and Chew, 2014). Providing literacy materials over the Internet accessed using mobile phones is a transformational opportunity for improvement of literacy levels in Africa. Nowadays, the use of mobile technology is becoming more common in development initiatives in Africa. It should be noted, however, that technology alone is not sufficient for sustainable development. The contents of the literacy programs need to be efficient to instruct accurate and fluent reading skill and then offer well-designed material which children are interested in approaching. Learning materials should be science-based and studied in the local environment by local people. Otherwise, the methodological flaws in current ways of literacy teaching are simply transferred onto the new platform.

In this article, we demonstrate how a scientific innovation originally targeted toward supporting a small minority in a high income Western country, has grown into a global endeavor for better literacy teaching and improved learning outcomes. With the help of internet and mobile technology, people around the world can benefit from this knowledge and adapt it to serve their own societies. This is the spirit of GG, to globally provide scientifically validated learning environment services that allow researchers to study the process of learning, teachers to learn better instruction methodology, parents to support their children’s education, and children to learn to read in a child-friendly game-based learning environment. The principles of proceeding in the provision of science-based eLearning services is the theme of the GraphoWORLD^3^ network of language, literacy, and learning scientists across the world.

## Field Testing GraphoGame in Africa

### Zambia

The story of GG in Africa began by providing a literacy game intervention for poor readers with 12 second-hand laptops in three public schools in Lusaka, late 2005 (for review, see Ojanen et al., 2013). The fact that the writing systems of Zambian languages (Kashoki, 1990) and of Finnish are both transparent, inspired us to test the possibility that the same intervention method for reading difficulties could work in both countries. Intervention results indeed showed increased performance in literacy tasks (Ojanen et al., 2013). Surprisingly, however, the study also revealed that children encountered extensive difficulties with vowels /a/, /e/, and /i/, the phonemes whose English letter names include vowel sounds that do not correspond with the vowel sounds of the these letters in the local Bantu languages (Ojanen et al., 2013; Ojanen, unpublished masters theses). It seemed that, while the language of initial literacy instruction had recently changed from English to local language with the New Breakthrough To Literacy program (Ministry of Education, 1996, 2003), the teachers were inadequately informed about the difference between the phonics of English and the phonics of Zambian languages and therefore continued to use English letter names in literacy instruction (Ojanen et al., 2013).

A pilot study in Zambia concluded that supporting children through provision of literacy training was possible although not sustainable unless teachers and parents were involved in the process. This was the premise for the Academy of Finland project Reading Support for Zambia (RESUZ), conducted by five Zambian Ph.D. students. The Project started in 2010 and by then, GG was already available for mobile phones and the intervention was provided to both the teachers and the learners. The medium of initial literacy instruction in the government schools of Lusaka was the widely spoken, local Bantu language, ciNyanja. Since the pilot study had suggested that teachers were not consistently teaching the correct letter-sounds in ciNyanja, a sub-study was conducted which assessed teachers’ knowledge of the sounds of the letters of local languages. The results revealed that teachers’ literacy skills varied significantly and some teachers had difficulties in letter-sound correspondences of ciNyanja, even though they were teaching literacy to first graders (Yalukanda, unpublished). It was therefore necessary to find a method that would support children’s literacy acquisition and also provide literacy instruction training to teachers.

The RESUZ intervention study was conducted with a representative sample of 573 Grade 1 learners in 42 Lusaka schools, and comprised a control group and various intervention combinations with the purpose of ascertaining the most efficient intervention model (Jere-Folotiya et al., 2014). Results from this field study conducted in low-resource government schools revealed that both direct and indirect exposure to the game improved reading acquisition, as documented by the literacy assessment data comparing children who had participated in the interventions to those in the control group. The best effect was obtained when both teachers and children were exposed to the game. Since GG was designed for early literacy skills practice for children, it was initially unprecedented that teachers also benefited from playing. After discovering that teachers had the tendency to instruct learners to recite the English letter names (Yalukanda, unpublished) rather than the ciNyanja letter sounds, it was understood that this could be one of the factors to explain why reading instruction was not more successful despite of use of local languages on Grade 1. This suggests that playing of the GG with even minimal pedagogical training and minimal or no experience in the use of ICT (information and communication technology), may enhance literacy teaching skills of first grade teachers and therefore support children’s learning opportunities in their classroom (Jere-Folotiya, 2014). This is most likely related to the teachers’ improved understanding of the correct phoneme pronunciation (Yalukanda, unpublished), and becoming aware of the incorrect cues given by the English letter names. This is in line with USAID finding that teachers have not received training in how to teach sounds of the letters during their pre-service training (Collins et al., 2012). The issue of teachers’ language and literacy proficiency requires more research and development of suitable assessment methods for teachers’ professional skills.

Moreover, the RESUZ project revealed the importance of parents’ attitude toward reading by studying a sub-sample of 72 low-income families with children participating in GG interventions (Chansa-Kabali, 2014; Chansa-Kabali et al., 2014). The study revealed that parents who displayed favorable reading attitudes engaged their children more in literacy practices at home, and that parental behavior correlated with the child literacy assessment results. Also, these parents provided more materials that enhance reading, such as children’s books (Chansa-Kabali, 2014). Thus, the results from RESUZ demonstrate how improving the quality of literacy instruction requires work on multiple levels: teachers, pupils, and parents. The use of ICT-based learning environment such as GG can benefit all these target groups in the future by guiding teachers to use more appropriate phonics in literacy instruction, by providing practice of decoding for children, and raising of awareness and literacy instruction for the parents.

The most recent literacy assessments from Zambia indicate that the literacy skills levels continue to be low. The empirical validation of GG in the local context of Zambian Government schools points to a viable opportunity to address this challenge. The Early Grade Reading Assessment (EGRA) baseline test conducted in 2012 (Rhodwell, 2013) by Read to Succeed Project reported that, under conditions of speeded testing, even minimal letter-sound knowledge and word recognition were absent in the vast majority of Grade 2 learners in rural areas. Out of 2000 Grade 2 learners that were assessed in oral passage reading, only 11% were able to read some words. The majority scored zero on letter-sound knowledge. Further analysis and comparison between EGRA and other literacy tests for validation of these results is currently in progress (Sampa et al., unpublished). In the context of the EGRA literacy assessment, the good news is that the University of Zambia is gaining momentum in provision of practical, evidence-based resources for addressing problems associated with instruction of reading skill. As soon as possible after acquisition of basic reading skill, it is important to encourage the children to acquire full literacy by providing them with exciting reading materials in local Zambian languages that motivate them to practice reading fluency and eventually help them to learn to comprehend even more demanding texts. The Centre for the Promotion of Literacy in Sub-Saharan Africa (CAPOLSA) was founded in 2011 with financial support from the Ministry for Foreign Affairs of Finland (Serpell, 2014) for this specific purpose. CAPOLSA promotes children’s literacy in Zambian languages and offers advanced education for young scholars to develop local expertise in learning related sciences.

**FIGURE 2:**
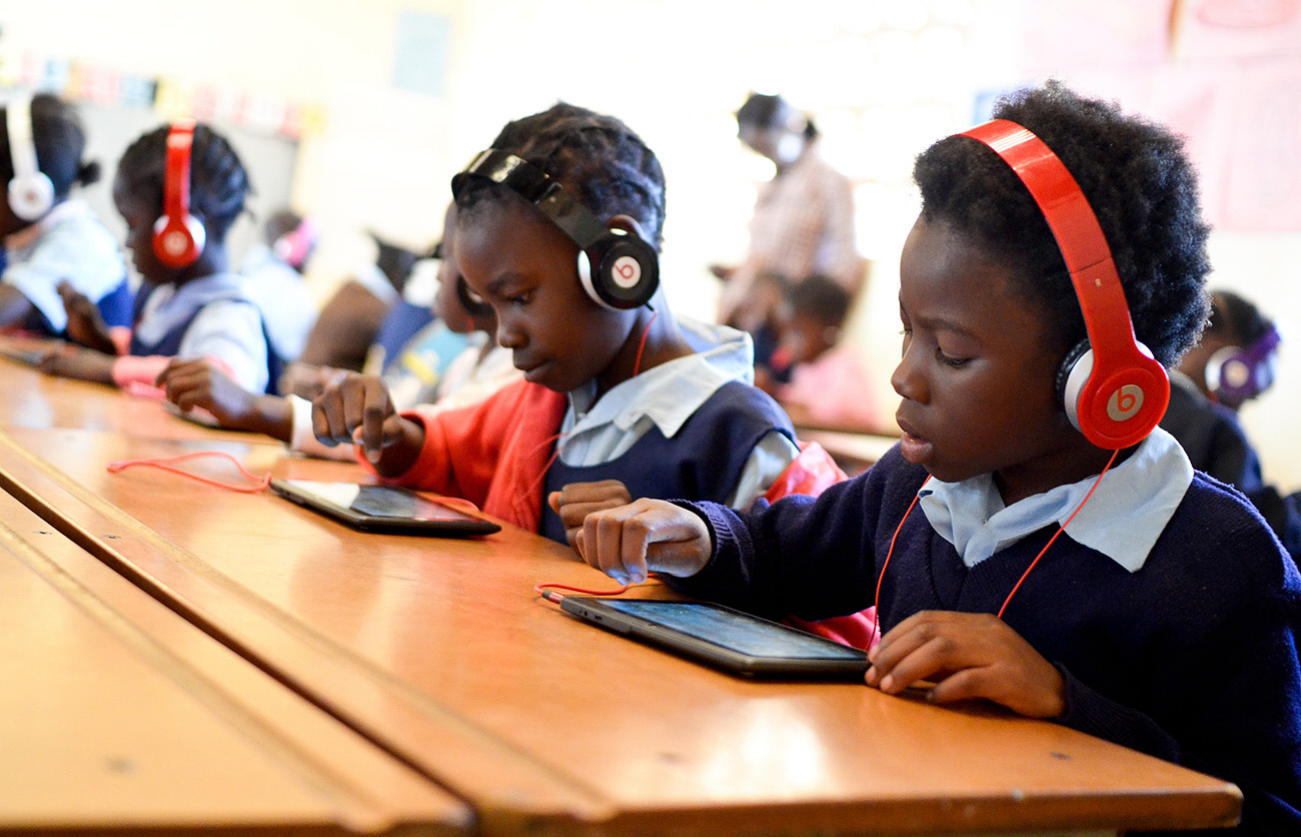
**Children playing with GG tablets in Lusaka 2013**. Picture credit Karri-Pekka Kauppinen/University of Jyväskylä.

The establishment of CAPOLSA and the fast progress of ICT becoming widely available have prompted new research questions. Now that we know that GG supports both teachers and children, we now need to know how best to distribute it to the learners in need. This was studied in Lusaka in February to August 2014 with the goal of finding the most effective method for providing GG exposure to the maximum number of first grade pupils in urban areas (Walubita et al., 2015). With six GG tablets per classroom, teachers were able to provide successful intervention for at least 30 children within a 2-months intervention period. This is a promising result since it was also confirmed that first grade children in Lusaka had problems in differentiating the vowels /a/, /e/, and /i/ during their intervention in 2013 (Kauppinen, unpublished masters theses). This suggests that those vowel confusions observed in 2005 (Ojanen et al., 2013) are still found in children’s GG game log records but also indicates that GG makes a significant impact on the acquisition of decoding skills by learners. In the future, current limitations of the GG experiments need to be addressed. Since the schools do not yet own tablets or phones for eLearning purposes, the only way to conduct the interventions is to provide the required equipment for the schools for limited time periods (see **Figure 2** as an example). It has not yet been possible to see what kind of learning benefits could be achieved if children had access to this technology every week throughout a complete semester, or used it regularly at home.

In 2013, the Ministry of Education revised the primary curriculum in order to expand the use of Zambian local language as the medium of instruction for Grades 2–4 and published a New Literacy Framework that emphasizes the use of phonics in early reading instruction (Sampa et al., unpublished). It is important that information concerning the new policy and available pedagogical guidelines is effectively disseminated to 1000s of teachers in Zambia, both in teacher training colleges and as in-service training to those already working in schools. With improved internet connections and high usage of mobile phones, it is expected that CAPOLSA can provide the required training by using the GG learning environment. In 2012, 76% of Zambians used mobile phone and 13.5% used internet (UNICEF, 2013a). Since the role of family involvement in urban Lusaka is already known, a new study (Ojanen et al., 2015) was conducted in October 2014 in rural Zambia where most parents have little or no formal education or no child-friendly reading materials at home. The aim of this study was to explore whether GG made available by lending a smart phone to Grade 2 children could also engage parents and other family members in literacy activities and improve their literacy skills. Fifty families living in the remote rural area of three Districts in Eastern Zambia participated in GG intervention with both a child and an adult member of the family using the game. The average playing time in the experiment was more than 5 h (compared to recommended minimum of 2 h) indicating that people were motivated to use eLearning environment for literacy practice. The environmental risk of poor literacy instruction can be helped with GG-based teacher training. The environmental risk of lack of community support can be helped by engaging parents in GG activities and disseminating literacy in local languages via mobile phones. With the new language policy for education, the hopes for improving the quality of education in Zambia are high.

### Kenya, Tanzania, and Namibia

Encouraged by the success of the Zambian GG team, the same approach has been introduced in Kenya, Tanzania, and Namibia. The first step of the GG adaptation process is the analysis of the existing language environment and current curriculum for literacy instruction. Local experts design the contents for the game and ensure the game’s effectiveness through controlled trials within an authentic environment. In Kenya, a validation study conducted in 2011 assessed the early literacy skills of Grade 1 children in Kikuyu and Kiswahili (Puhakka et al., 2015). The challenge in Kenya is that, while Kiswahili, which has a relatively transparent orthography is used as the medium of instruction, it is actually not the principal medium of communication in the majority of children’s homes. The results showed that the children who received the intervention of at least 4 h training time improved in their orthographic awareness in Kikuyu and Kiswahili. Another study with 128 GG Kiswahili players showed improved letter-sound knowledge, word recognition, pseudoword reading, and single word spelling (Puhakka et al., 2015). These results indicate that GG can provide effective support in Kenya, where currently, most teachers lack the necessary training to teach early reading skills. Moreover, since it may take a long time to create learning contents for all African languages, it is positive that children who are learning Kiswahili as their second language were also able to benefit from GG. Use of GG may also help to promote the use of Kiswahili and other Kenyan languages in education.

Even though Tanzania has been celebrated for its success in promoting a national language and literate culture in an African language and literacy among children and adults (Alidou, 2011), there is still room for improvement as 20% of the adult population is illiterate (UNESCO/UNICEF, 2013; UNICEF, 2013b). An experimental study was conducted in Bagamoyo in 2014 by the GG research team at Sebastian Kolowa Memorial University to establish the efficacy of GG in supporting the acquisition of adequate basic skills for learning to read. The preliminary data analyses of the pilot study suggest that, while providing teachers with training in phonics-based literacy instruction is important and has a significant effect on learning, the outcomes improve more when GG is provided for the learners. The overall goal for the future is to use the GG Kiswahili as an intervention method to improve children’s acquisition of basic skills for reading and thus minimize the risk of developing persistent reading difficulties. Similar to Zambia, GG activities in Tanzania, include training teachers in synthetic phonics-based instruction methods, creating reading materials in Kiswahili and providing expertise in curriculum development. The GG research team at SEKOMU is conducting a large study in seven intervention schools and three control schools in March 2015.

Adaptation of GG in Namibia has proceeded to expand the use of the GG learning game in teacher education units as a method to instruct effective use of phonics. Furthermore, the GG content has also been extended to train basic mathematics in Namibia. Similar to many other African countries, Namibia is plagued by a large portion of its learner population being unable to read according to standard. According to SACMEQ reports I–III and EGRA pilots (Gains and Parkes, 2012), Namibia has been consistently ranked near the bottom. Forty-percent of Grade 5 learners only manage to read at the Pre-Reading, Emergent Reading, or Basic Reading Levels (Hungi et al., 2010). Therefore, solutions to improve early grade literacy are in great demand in Namibia. GG was implemented to Afrikaans and, at the same time, to follow the literacy curriculum in Grades 1 and 2. In the first validation study in 2013 by Pamela February at the University of Namibia, 83 first grade learners played GG Afrikaans literacy game, 80 played GraphoMaths and 40 learners acted as non-playing controls. This study showed that learners who played GG Afrikaans for 300 min (5 h) performed significantly better in post-assessment literacy assessment tests compared to controls. Follow-up study in 2014 provided additional 10 h long GG Afrikaans training to a selected 19 learners who participated in the earlier study but who did not improve sufficiently. Preliminary results indicate that longer exposure to the GG also improved letter-knowledge, phonological awareness, and reading skills of these struggling readers. In addition to the GG validation study, the team at the University of Namibia is involved – as mentioned above – in developing GG interventions and eLearning material for teacher training purposes in collaboration with the Ministry of Education and UNESCO.

### E-Learning Environments for Teachers

Improving literacy in Africa is not only about the need to build academic capacity and scientific expertise around learning-related sciences. This knowledge needs to be disseminated to the correct stakeholders. The central role that teacher educators play in shaping teachers’ skills is often the most neglected aspect of teacher preparation systems (Education for all Global Monitoring Report [EFA GMR], 2013/2014). The University of Jyväskylä and Niilo Mäki Institute have been involved in training the teacher educators for over two decades in several projects in Africa. The focus of these projects has been to provide the latest information on neuropsychology and learning difficulties to the key individuals who work as lecturers in learning-related disciplines or who participate in policy making in the Ministry of Education. It is indeed difficult to imagine how the quality of teacher training could be improved unless there is a sufficient number of local experts who are professionally capable of training teachers to teach literacy and also to identify and support children with learning difficulties, as recommended by UNESCO (Education for all Global Monitoring Report [EFA GMR], 2013/2014).

In 2012, the Niilo Mäki Institute started a literacy training program in Zambia, Namibia, Kenya, and Tanzania for teacher educators. The main goal of this program was to train literacy specialists who have the latest evidence-based knowledge of learning to read and reading difficulties. GG technology was utilized in this project as the main tool to support learning to read. The 3-years “GraphoLearn Diploma Training Program” was funded by the Ministry of Foreign Affairs of Finland. The 25 trainees and their four local trainers participated in two annual workshops. Each workshop focused on different aspects of emergent literacy, for example, grapheme–phoneme correspondences in transparent languages, phonological awareness, decoding skills, reading fluency and reading comprehension. Assessment and literacy case-study interventions using GG were also included in the program. In every workshop, the trainees presented the results of the case studies that they had carried out in their own countries between the workshops. In these small intervention studies, they practiced how to teach literacy and how to use GG as a part of the training. The local trainers supervised these interventions and reported them to the Finnish trainers. The trainees have learned to identify, assess, and train reading difficulties and as teacher educators, they can pass on these new skills to their students and to the curricula of their universities and teacher training colleges.

As mentioned in Education for all Global Monitoring Report [EFA GMR] (2013/2014), it is not only the quality of teacher education that is insufficient. Another problem is the lack of capacity to train the huge numbers of teachers. The use of mobile technology, particularly in low-income countries has the greatest potential for ICT-based learning and allows large numbers of trainees (Education for all Global Monitoring Report [EFA GMR], 2013/2014) to be reached. This is why we are directing our efforts toward the creation of an eLearning environment for teachers and teacher trainers to facilitate better literacy instruction. The eLearning material will contain both theory and practice, exercises and video clips on best practices to teach reading using local languages in different African countries. The material will also include sample texts and stories to support and assist both teachers (to assess children’s reading progress) and children (to practice their acquired reading skill). A special module will be included on reading difficulties and how teachers can identify and assess such difficulties. This will guide teachers on how to teach children with additional support needs within an inclusive environment. The theoretical materials will be in English and the more practical examples and exercises in various local languages used in literacy training in partner countries. The outcome of the project will be similar to a compact comprehensive eLearning book for teachers. The materials will be freely accessible online and can be used in various electronic devices such as smartphones, tablets, laptops, and PCs or used in other digital format (e.g., DVDs and memory sticks or similar). Teachers can self-study materials about literacy instruction and learning difficulties and their learning can be monitored over the internet by CAPOLSA experts. Teachers can then have access to science-based information on education, and most importantly, information that is produced by local experts in education sciences.

## Discussion

Quality of education and ensuring literacy acquisition in early grades are fundamental themes in the global post-2015 agenda. One key area is the need for knowledge on inter-dynamics between language and literacy. Conducting interdisciplinary research, consensus building and awareness-raising campaigns to update knowledge on language in education and development is recommended (Ouane and Glanz, 2010). This work begins with acknowledging general conclusions of the previous research (Ball, 2011), such as the importance of their first language to children’s cognitive development and academic achievement and ensuring that children become highly proficient in one language (which takes 6–8 years) before requiring academic work in a second language. As our examples from GG activities in Africa demonstrate, provision of efficient literacy instruction, even for early grades in local African language, is still a massive challenge. Lack of scientific knowledge on language development, literacy acquisition, and learning in general is a rarely mentioned challenge in the area of international development and yet, it is difficult to see how teacher training programs or production of learning materials could improve without a solid foundation in science. The GG technology, based on intensive longitudinal literacy research of children at risk for dyslexia in Finland (for a recent review of the results, see Lyytinen and Richardson, 2014), allows fast data collection and use of comparable research methods across the countries. Better still, the GraphoWORLD network allows scientists to join forces with colleagues across the world and probably most importantly for Africa, to develop inter-regional collaboration between countries that are struggling with similar challenges. In conclusion, GG is more than a mobile application for practicing basic literacy skills. Adaptation of the game to new countries and language environments has a much larger impact on the education sector as a whole, prompting the need for upgraded policy making, curriculum development, and teacher training. Training African scholars and providing them with cutting-edge research technology empowers them to take their places as qualified experts and advisers that policy-makers need for making informed choices for better education quality.

Education for all Global Monitoring Report [EFA GMR] (2015) states that language is of considerable importance for the quality of teaching and learning but among the biggest obstacles to using local languages in the classroom are a lack of textbooks and a shortage of trained teachers using the languages. The use of mobile technology, particularly in low-income countries has the greatest potential for ICT-based learning and allows large numbers of trainees (Education for all Global Monitoring Report [EFA GMR], 2013/2014) to be reached. But striving for better literacy knowledge among scholars, teacher educators, and teachers is not yet enough for sustainable impact. Research on GG has also revealed the need to engage parents and larger community in literacy activities. This is in line with the recommendation to position parents and other family members, as well as the whole community in planning, implementation, and evaluation of literacy programs (Ball, 2011). It has been shown that parental attitude toward children’s reading practices had a significant impact on the learning outcomes in both Zambia and Tanzania (Ngorosho, 2011; Chansa-Kabali, 2014) and that a major challenge to increasing the use of local languages in education is the negative attitude toward African language at home (Chansa-Kabali, 2014; Kwena et al., 2014; Puhakka et al., 2015). In Kenya, 41% of the teachers reported that parents were against their children being taught early reading skills in indigenous languages and 77% indicated that parents prefer that their children are taught early reading skills in English (Puhakka et al., 2015). In Kenya, misinformation about language policy among parents and teachers is the major cause for failing to implement the language policy for teaching literacy in local language (Kwena et al., 2014). However, there is evidence that supporting children’s reading in the mother tongue also inspires their parents to learn to read and participate in adult literacy courses (Ouane and Glanz, 2010). Thus, the process of identifying weak learners at school and providing them with literacy intervention using a mobile phone can have a wide impact on the literacy levels of the whole community.

In September–October 2014, CAPOLSA included a teacher-orientation component in the design of the latest GG field test in rural areas (Ojanen et al., 2015) and conducted a pilot study on a mobile phone-based teacher training program. This involved training the teachers about the new phonics-based curriculum and providing them with GG and related video materials for 5 weeks. Teachers gave selected pupils GG mobile phones to be used either at school or at home. Mobile-based teacher in-service training could be a cost-effective way for teachers to update their literacy teaching skills and provide much required support for struggling learners and, at the same time, educating the parents about literacy issues. Parents and the local community could then be encouraged to create their own literacy materials. This was already tried in Zambia with the Kalulu writing competition for local languages. Stories from the competition are now used in eLearning materials on CAPOLSA^4^ tablets and phones.

Even though we are currently able to provide mobile-based literacy intervention to teachers, children, and parents, and thus increase the knowledge on reading acquisition in Africa, there are still many challenges. Some studies report that, although ICT seems to be a promising tool in an educational context, many teachers are reluctant to integrate it in their daily practice, even in the developed countries (Kreijns et al., 2013; Van Acker et al., 2013). In developing countries, limited technical resources (e.g., lack of devices, electricity, internet connections) and support are still extra barriers for use of ICT, even though 3G and 4G networks are developing fast and the prices of smartphones and tablets are also decreasing rapidly. When we try to boost teachers to use ICT to facilitate learning, we know that some degree of change is required in the following dimensions: (a) beliefs, attitudes, or pedagogical ideologies (e.g., a commitment to empirical discovery of the best methods of literacy learning), (b) content knowledge (e.g., theory of literacy learning), (c) pedagogical knowledge of instructional practices, strategies, methods, or approaches (how to teach reading and how to learn to read via proper instruction and using GG technology), and (d) novel or altered instructional resources (e.g., smartphones or tablets). Despite all the remaining challenges, UNESCO hopes that mobile reading will be integrated into broader educational systems that teach people how to use text productively – from access to comprehension, and all the stages in between (West and Chew, 2014).

Another major challenge is to ensure that learners practice for sufficient time. By all means, this should mean long enough to attain reading fluency, which is required before the learner is ready to transition to the use of new languages (Abadzi, 2013). Fluency does not develop without a sufficient quantity of engaging and motivating reading materials. Combining GG with science, technology, engineering, and mathematics learning materials is one option for providing more literature for learners. The aim for the next GG studies should be to ensure reading fluency in early grades and to lay the foundations for reading comprehension.

Distribution of GG and related eLearning content is another long-term process which will most likely involve collaboration with governments of the local countries, as well as support from non-governmental international organizations and private companies. The preparation of GG commercialization has been supported by Tekes, The Finnish Funding Agency for Innovation, in a research project called “Grapho Learning International Development and Exports” which was conducted during 2012–2015. While the adaptations of the new game versions can be conducted as research projects, or within international development projects, the provision of user support in the long run requires other solutions. The next challenge is to expand the GG technology-enhanced support globally. Currently, GG utilizes a project-based delivery model consisting of public-private partnership and donor funding to enable development of the evidence based learning tool in different languages and parts of the world. While approaching new countries and languages, development of a new kind of business model is necessary to ensure financial sustainability and scalability of the initiative. These plans are under development but the aim is to complement the long-term public procurement and donation model by establishing a company and contracting with local distributors. This could mean for example the development of new language versions in collaboration with GraphoWORLD member universities, licensing and distributing the GG service and analytics by the company, and certifying local partners to operate the related training and support services.

The aim of GG activities should be to provide services free of charge to the end user in the developing countries. From the business model perspective, the customer could be the adult caretaker of the learner, school, or government, depending on the market situation in a given partner country. GG related research has laid the foundation for the next step for improvement of global literacy. This step, purporting to support dissemination, includes all the processes necessary to make the public procurement or other distribution mechanism possible in each country that wants to make the GG service available in one form or another. Such a step can be taken as the IPR protection (patenting), branding (trademarking), and stakeholder management (communication plan) have been carefully prepared to include the GraphoWORLD network of researchers in the world’s top-universities, ministries, funding partners and sponsors, as well as the general public, in order to raise awareness of the literacy levels and potentially to make a difference by providing inclusive literacy learning for all.

This new information on learning needs then to be disseminated to the right stakeholders. Policy makers and teacher educators need to be informed about the new evidence-based methodologies. GG teams in Africa have been active in discussions with their Ministry representatives and have provided expert consultation for curriculum development in teacher training colleges and formulation of new literacy instruction guidelines. If and when the teacher training curriculum or literacy instruction materials change, there is a need to inform 1000s of in-service teachers about these changes. Creating eLearning environments for teachers is a way of disseminating this information very quickly and GG technology allows monitoring of teacher’s professional capacity. One way to do this is to monitor teachers’ literacy and language skills from the GG game logs and a certificate can be awarded when they have reached adequate skill level. Certificate can be given if the teacher shows adequate knowledge of local language literacy by playing GG and is able to successfully organize a GG literacy intervention in her or his classroom and increase the children’s literacy skills with sufficient amount of practice. Performance in the game can be monitored over the internet. This GG instruction certification design was developed by the University of Zambia–CAPOLSA research team in close consultation with the Zambian Ministry of Education, Directorate of Teacher Education and Specialized Services (TESS; Walubita et al., 2015) and it is being developed further in order to create a mobile-based teacher training service for teachers’ professional development and in-service training. The focus of the interventions are the children who are struggling with literacy and falling behind their peers in the classroom. African children with learning difficulties need support and it has already been shown that playing GG benefits children, even in large classrooms where the teacher is already strained with work (Jere-Folotiya, 2014). Providing GG at home can engage parents and other caretakers in literacy activities. This is needed to raise awareness about the importance of local languages and also to improve adult literacy and help to dispel the negative attitude toward African language at home (Chansa-Kabali, 2014; Kwena et al., 2014; Puhakka et al., 2015).

In summary, GG can contribute toward addressing the global literacy crisis on multiple levels. It is apparent that any changes in teacher training, curriculum development, policy making, and production of learning materials needs to be based on solid science and local academic expertise. If the international community wants Sub-Saharan countries to improve their quality of education, it is necessary to provide African scholars and academic institutions with adequate resources and support to conduct research on language, literacy, and learning related topics. For the scientific community, GG is a research laboratory in a mobile phone, enabling much needed research on the process of literacy acquisition. For the teachers, GG can provide relief in their daily struggle with multiple problems. For policymakers, GG provides an opportunity to articulate concrete connections among language, orthography, curriculum, teaching materials, and effective literacy instruction for the next generation of children. While there are many long-term, macrosocial, intertwined challenges for developing education in Africa, there is something that we can do immediately and with little cost: scientific information on learning needs to be available and accessible to all teachers. Without scientific knowledge, all other attempts to improve education are likely to fail expectations. Brand new school buildings and higher salaries have little effect if the teachers never receive the pedagogical training for their work.

Sustainable improvement in the quality of education and literacy learning outcomes is a process which needs to happen on multiple levels at the same time: planning education policy, developing learning materials for schools, providing teachers with evidence-based pedagogical training, engaging parents in literacy practices and supporting children with reading difficulties. All this requires scientific research on literacy learning process in authentic environments in local languages by local people. GG and GraphoWORLD network can facilitate this process, not only in Africa, but elsewhere in the world. The latest development of this process is the establishment of the UNESCO Chair on Inclusive Literacy Learning for All, under the leadership of Professor Heikki Lyytinen, the inventor of GG. The purpose of the UNESCO Chair is to promote evidence-based education policies, scientifically valid instruction methods, and effective teacher training programs. Mobile phones can provide this information for teachers and other education professionals around the world. GG can both support the children with learning difficulties and collect data for further research on language, literacy, and learning. Thus, a simple learning game designed for a small minority of Finnish children can now provide learning opportunities for struggling readers across the globe.

## Author Contributions

EO is a project researcher at the Agora Center, University of Jyväskylä and is writing her Ph.D. in Psychology on learning letter-sound correspondences in Zambian languages.

MR has worked as a post-doctoral researcher at the Agora Center, University of Jyväskylä and has studied the motivational and learning effects of GraphoGame since early 2000s.

TA is Professor at the Department of Psychology, University of Jyväskylä and has been working in Niilo Mäki Foundation international cooperation projects in Africa since 1990s.

TC-K is a lecturer at the Department of Psychology, University of Zambia, was part of the Reading support to Zambia-project and wrote her Ph.D. on the effect of home environment on children’s literacy skills.

PF is a lecturer at the Department of Psychology, University of Namibia and she is writing her Ph.D. on the use of GraphoGame in practice of literacy skills in Afrikaans.

JJ-F is a lecturer at the Department of Psychology, University of Zambia, was part of the Reading support to Zambia-project and wrote her Ph.D. on teaching practices of literacy and use of GraphoGame in Zambia.

K-PK worked at the Agora Center, University of Jyväskylä and wrote his Master’s thesis in Psychology on the letter-sound error patterns of the 1st grade GraphoGame players in Zambia.

RK is a lecturer at the Department of Teacher Education, University of Helsinki, has been working in Niilo Mäki Foundation international cooperation projects in Africa since 2001 and is part of the original Finnish Ekapeli development team.

DN is a lecturer at the Sebastian Kolowa Memorial University, Tanzania and has been been involved with GraphoGame research since 2010.

MP is the head of the project management at the Agora Center and has been working in Africa GraphoGame projects since 2008.

SP is a project researcher at the Agora Center, University of Jyväskylä and wrote her Ph.D. on the use of GraphoGame in Kiswahili and Kikuyu languages in Kenya.

FS is working at the USAID/Read to Succeed project in Zambia, was part of the Reading support to Zambia-project and writing his Ph.D. on children’s early literacy skills in Zambia.

GW is a lecturer at Department of Educational Psychology, Sociology and Special Education, University of Zambia, and has conducted research on the use of GraphoGame on tablets in Zambian classrooms.

CY is working at the Zambian National Union of Teachers, he was part of the Reading support to Zambia-project and writing his Ph.D. on teachers’ literacy skills and knowledge of literacy instruction methodology for Zambian languages.

KP is a Head of the Haskins Laboratories, Professor at Yale University and a member of the GraphoWORLD network of excellence board.

UR is a Professor at the Agora Center, University of Jyväskylä and is the head of the GraphoGame unit.

RS is a Professor at the Department of Psychology, University of Zambia and is the head of the CAPOLSA.

HL is the inventor of the GraphoGame and holds the UNESCO Chair on Inclusive Literacy Learning for all, coordinated by the Agora Center, University of Jyväskylä.

## Conflict of Interest Statement

The authors declare that the research was conducted in the absence of any commercial or financial relationships that could be construed as a potential conflict of interest.

## Footnotes

1 http://info.graphogame.com/

2 http://www.lukimat.fi

3 http://info.graphogame.com/wp-uploads/2015/02/GraphoWORLD-Declaration_2010_2015.pdf

4 http://humanities.unza.zm/index.php/departments/social-development-studies/history/136-childrens-stoires
